# Gut microbiome composition and diversity are related to human personality traits

**DOI:** 10.1016/j.humic.2019.100069

**Published:** 2020-03

**Authors:** Katerina V.-A. Johnson

**Affiliations:** University of Oxford, Department of Experimental Psychology, New Radcliffe Building, Radcliffe Observatory Quarter, Oxford OX2 6GG, United Kingdom

**Keywords:** Gut microbiome, Microbiome–gut–brain axis, Metagenomics, Human personality, Social behaviour

## Abstract

The gut microbiome has a measurable impact on the brain, influencing stress, anxiety, depressive symptoms and social behaviour. This microbiome–gut–brain axis may be mediated by various mechanisms including neural, immune and endocrine signalling. To date, the majority of research has been conducted in animal models, while the limited number of human studies has focused on psychiatric conditions. Here the composition and diversity of the gut microbiome is investigated with respect to human personality. Using regression models to control for possible confounding factors, the abundances of specific bacterial genera are shown to be significantly predicted by personality traits. Diversity analyses of the gut microbiome reveal that people with larger social networks tend to have a more diverse microbiome, suggesting that social interactions may shape the microbial community of the human gut. In contrast, anxiety and stress are linked to reduced diversity and an altered microbiome composition. Together, these results add a new dimension to our understanding of personality and reveal that the microbiome–gut–brain axis may also be relevant to behavioural variation in the general population as well as to cases of psychiatric disorders.

## Highlights

• Investigation of gut microbiome composition and diversity with respect to human personality.
• Analyses targeted bacterial genera linked to behaviour in animal and human psychiatric studies.
• Bacterial genera were modelled (using negative binomial regression) with respect to personality.
• Genera linked to autism are also related to social behaviour in the general population.
• Sociability is associated with higher diversity, and anxiety and stress with reduced diversity.

## 1 Introduction

Personality shapes our world. It influences our health, our friendships, how we deal with stress, what jobs we succeed in and how we like to spend our time. It is approximately 50% heritable in human populations [1], suggesting that environmental factors also contribute significantly to our personality. In addition to our external environment, the burgeoning field of the microbiome is revealing the many ways that our ‘environment within’ can affect our body’s physiology, including our digestion, immunity, metabolism, development and even our behaviour [2], [3], [4], [5], [6]. In fact, the number of microbial cells in our bodies is estimated to be roughly equal to our own human cells [7], with the majority of these microorganisms inhabiting the gut. Studies in the past decade provide evidence that the gut microbiome interacts with the central nervous system [8] and such findings have the potential to aid the development of new treatments for conditions such as autism [9] and depression [10]. However, research has largely been conducted in animal models and it is unknown how translatable these findings are to humans [11], [12], [13]. While there is some indication that dysbiosis of the gut microbiota may be implicated in neurological and psychiatric disorders [14], an open question remains as to whether variation in the gut microbial community is related to personality, that is behavioural differences between individuals that are consistent over time and different situations and therefore broadly predictable.

Animal studies have demonstrated that the gut microbiome can influence the stress response, anxiety and depressive-like behaviours, as well as social behaviour and communication [6], [15]. Some of the most convincing findings stem from faecal microbiota transplantation whereby behavioural traits can be transferred between mouse strains when their gut microbiota are swapped [16], [17]. For example, when the more anxious and timid Balb/c mice are colonized with the gut microbiota of NIH Swiss mice, their temperament becomes more bold and exploratory like that of the donor NIH Swiss mice, and vice versa [16]. Further support comes from the induction of anxiety and depressive-like behaviours in rodents colonized with the gut microbiota of humans suffering from these symptoms [18], [19], [20]. The transmission of such behaviours via the microbiota therefore suggests that gut microorganisms can contribute causally to behavioural traits. In fact, a recent human study reported improvement in psychiatric symptoms following faecal microbiota transplantation in patients with gastrointestinal disease [21].

There are numerous possible mechanisms that may mediate this interaction between the gut microbial community and the brain, including communication via neural, immune and endocrine pathways [6], [22], [23]. Microorganisms can also produce various neuroactive chemicals [24], [25] and can modulate host neurotransmitter levels [26]. For example, gut bacterial species such as those belonging to the genus *Bacteroides* have been shown to produce γ-aminobutyric acid (GABA) in large quantities in culture [27]. More recently it has been reported that the relative abundance of *Bacteroides* is negatively associated with brain signatures of depression [28], suggesting that bacterially derived GABA may play a role in the microbiome–gut–brain axis. Gut dysbiosis might lead to imbalances in neurotransmitters, inflammation or heightened activity of the hypothalamus–pituitary–adrenal axis that regulates the stress response [13]. The observation that psychiatric illnesses are often comorbid with gastrointestinal problems [29], [30] supports the role of the microbiome–gut–brain axis in human biology and psychology. A number of studies has reported associations between the composition of the gut microbiome and conditions such as autism, depression and schizophrenia (Table 1). However, results sometimes differ with regard to changes in the abundances of specific bacterial taxa, likely due to individual variability in gut microbiome composition together with underpowered studies. Furthermore, there is limited understanding of the mechanisms via which each of these bacterial taxa may affect the brain. Indeed, the predominant mechanism likely varies depending on the particular taxon involved, including anti-inflammatory effects, the production of short chain fatty acids, hormonal effects, the release of neuroactive metabolites including neurotransmitters and stimulation of the vagus nerve [23].

**Table 1:** Summary of statistically significant associations between bacterial genera in the gut and behavioural or psychiatric traits, as reported in the literature. Table lists findings from research in both animal models and human populations and includes all genera (23 in total) associated with these traits in at least two independent studies.

Genus	Change in abundance	Behavioural trait/psychiatric condition	Study subject	References
*Akkermansia*	↓	Autism	Children	[52]
	↑	Autism	Children	[53]
	↓	Stress	Mice	[54], [55]
*Alistipes*	↓	Autism	Children	[56]
	↑	Stress	Mice	[57]
	↑	Depression	Adults	[58], [59]
*Bacteroides*	↑	Autism	Children	[53], [60]
	↓	Stress	Mice	[55], [61]
	↑	Negative mood	Adults	[62]
	↑	Psychosis	Adults	[63]
*Bifidobacterium*	↓	Autism	Children	[52], [53], [60], [64], [65], [66], [67]
	↑	Autism	Children	[68]
	↓	Autism	Mice	[69]
	↓	Stress	Mice	[70], [71]
	↑	Stress	Mice	[72]
	↑	Negative mood	Adults	[62]
	↓	Depression	Adults	[73]
*Blautia*	↓	Autism	Infants	[74]
	↓	Autism	Children	[75]
	↓	Autism	Mice	[69]
	↓	Schizophrenia	Adults	[76]
*Clostridium*	↑	Autism	Children	[53], [56], [67], [75], [77], [78], [79], [80]
	↓	Autism	Children	[60]
	↑	Stress	Mice	[61], [72], [81]
	↑	Depression	Adults	[59], [82]
	↑	Schizophrenia	Adults	[76]
*Collinsella*	↑	Autism	Children	[56]
	↓	Autism	Children	[60]
	↑	Schizophrenia	Adults	[76]
*Corynebacterium*	↑	Autism	Children	[56]
	↓	Stress	Rats	[83]
*Desulfovibrio*	↑	Autism	Children	[60], [80]
*Dialister*	↓	Autism	Children	[56], [60]
	↑	Sociability	Infants	[84]
	↓	Depression	Adults	[18], [59], [85]
*Dorea*	↑	Autism	Children	[53], [56]
	↓	Autism	Children	[75]
	↓	Stress	Mice	[61]
*Faecalibacterium*	↑	Autism	Infants	[74]
	↓	Autism	Children	[53], [86]
	↓	Negative mood	Adults	[87]
	↑	Negative mood	Adults	[62]
	↓	Depression	Adults	[59], [85]
	↑	Depression (prenatal)	Adults	[88]
	↓	Bipolar disorder	Adults	[89], [90]
*Flavonifractor*	↑	Autism	Children	[75]
	↑	Depression	Adults	[59]
*Lactobacillus*	↑	Autism	Children	[56], [64], [68], [80], [91]
	↓	Autism	Children	[53]
	↓	Stress	Mice	[61], [72], [92]
	↓	Stress	Rats	[83]
	↓	Stress	Macaques	[93]
	↓	Depression	Adults	[73]
	↑	Psychosis	Adults	[63]
*Lactococcus*	↓	Autism	Children	[53], [60]
*Oscillibacter*	↑	Depression	Adults	[58]
	↑	Stress	Rats	[83]
*Oscillospira*	↓	Autism	Children	[53]
	↑	Sociability	Mice	[94]
	↓	Stress	Mice	[55], [94], [95]
*Parabacteroides*	↓	Autism	Children	[56]
	↑	Autism	Children	[60]
	↑	Sociability	Infants	[84]
	↓	Stress	Mice	[55], [61], [92], [94]
	↑	Negative mood	Adults	[62]
	↑	Depression	Adults	[59]
*Prevotella*	↑	Autism	Children	[53]
	↓	Autism	Children	[91]
	↑	Stress	Mice	[94]
	↓	Negative mood	Adults	[62]
	↑	Depression	Adults	[82]
	↓	Depression	Adults	[18]
*Roseburia*	↑	Autism	Children	[53]
	↑	Stress	Mice	[61]
	↓	Stress	Mice	[55]
	↓	Negative mood	Adults	[62]
	↓	Schizophrenia	Adults	[76]
*Streptococcus*	↓	Autism	Children	[53], [60]
	↑	Depression	Adults	[82]
*Sutterella*	↑	Autism	Children	[52], [65], [96], [97]
	↓	Autism	Children	[75]
	↑	Stress	Mice	[55]
*Turicibacter*	↓	Autism	Children	[53]
	↑	Sociability	Mice	[94]
	↓	Stress	Mice	[70], [72], [94]
	↑	Depression	Adults	[18]

Intervention studies in humans have started to investigate whether the gut microbiome makes a detectable contribution to the functioning of the central nervous system. Although studies administering probiotics have yielded mixed results in humans, meta-analyses do provide support for their beneficial effect on stress, anxiety and depressive symptoms, including in healthy humans as well as those suffering from psychiatric conditions [31], [32], [33], [34]. In addition, brain imaging studies have revealed that consumption of probiotics in healthy volunteers can affect neural signatures of emotion [35], [36]. For example, probiotic administration over a period of four weeks was found to modulate brain activity, resulting in reduced activity of brain regions involved in emotional processing when participants were presented with negative facial expressions [35]. In studies involving prebiotics, healthy humans show a reduction in salivary cortisol levels after three weeks of taking the supplement [37] and patients with irritable bowel syndrome have reported reduced anxiety following one month of prebiotic supplementation [38].

Since the gut microbiome has been implicated in social development and behaviour [39], [40], [41], [42], recent research has investigated whether it may modulate behavioural symptoms of autism [43]. Autism is not only characterized by impaired social behaviour but is frequently comorbid with gastrointestinal issues [44] and immune dysfunction [45], [46]. Studies comparing autistic individuals with neurotypical controls often report significant differences in gut microbiome composition (Table 1). Interestingly, a trial of faecal microbiota transplantation in children with autism found that it improved not only gastrointestinal symptoms but also social behaviour and communication [47], including two years after completion of the treatment [48]. This suggests that the gut microbial community may play a part in the expression of autistic behavioural traits. Given the evidence that autistic traits are normally distributed across the population [49], [50], [51], gut microbiome composition may also be related to variation in sociability in the general population.

The main aim of this research was to determine whether individual variation in the composition and diversity of the human gut microbiome is related to differences in personality. Previous research has focused on animal models or examined broadscale associations between microbiome composition and psychiatric symptoms. In contrast, here targeted regression analyses were conducted to investigate the relationship between personality traits and the abundances of specific bacterial genera identified in the microbiome–gut–brain axis literature (Table 1). It is hypothesized here that genera previously associated with autism may be related to differences in sociability in the general population, while genera previously linked to depression and stress in animalmodels or psychiatric populations may be differentially abundant in the general population with respect to neurotic traits. As well as assessing genus-level composition, the relationship between personality and measures of microbiome diversity was also investigated, with the hypothesis that variables assessing sociability may be positively related to diversity and those assessing neurotic traits may be negatively related.

## 2 Materials and methods

### 2.1 Study population

**Fig. 1:**
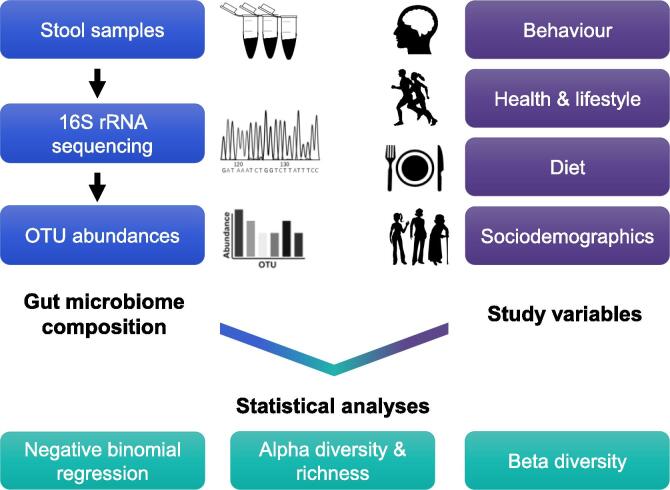
Overview of study design. A faecal sample was provided by each participant using the commercially available uBiome Gut Kit and sample processing and sequencing were carried out by uBiome to generate OTU (operational taxonomic unit) abundances. Participants were asked to complete a comprehensive study questionnaire assessing their behaviour, diet, health, lifestyle and sociodemographics (see Fig. S1 for variables tested within each of these categories). A range of statistical analyses was conducted to determine the relationships between gut microbiome composition and the study variables, with a primary interest in the variables assessing behavioural traits.

Adults (over 18 years) were invited to take part in the study (Fig. 1). A faecal sample was received from 671 individuals but sixteen samples were removed (*n* = 655) since they did not meet the read quality control threshold set at 10,000 reads to ensure detection of low abundance taxa [98]. The research was conducted under a Human Subjects Protocol provided by an institutional review board (Ethical and Independent Review Services, IRB Study #13044-03). All participants provided informed consent and data were analysed anonymously. The sample was 71% female and 29% male, with a mean age of 42 years. Participants resided in twenty different countries and four continents, predominantly North America (83%), with participants from USA accounting for 77% of the sample. 11% of the study participants were from Europe (4% from UK), 5% from Australasia and 2% from Asia.

### 2.2 Sample collection, processing and sequencing

The microbiome sequencing company uBiome provided participants with their commercially available Gut Kit. Each participant was supplied with one kit to be used for faecal sample collection at home. Analysis of faecal samples is the standard procedure in microbiome research since they are sufficiently representative of the composition of the gut microbiome [99]. The sampling procedure followed the guidelines of the National Institutes of Health Human Microbiome Project [100]. The sample collection tube contained a lysis and stabilization buffer for DNA preservation during transport at ambient temperatures [98]. The 16S rRNA gene was amplified using universal primers for the V4 hypervariable region and sequencing was performed on the Illumina NextSeq 500 platform, alongside positive and negative controls. Standardized methodology [98] for sample processing and sequencing was conducted by uBiome and has been shown to generate reproducible microbiome profiles [101].

### 2.3 Questionnaires

An online questionnaire designed for this study was distributed to participants. In total, 44 study variables (continuous and categorical) were analysed relating to behavioural traits, diet, health and lifestyle and also sociodemographics. While the majority of variables were assessed through binary questions or point scales, personality and social behaviour were evaluated with widely used and validated questionnaires from psychological research. The 50-item International Personality Item Pool [102] was used to assess personality traits according to the five-factor model of personality. A large number of studies find that individual differences in human behaviour can broadly be divided into five key domains, known as the ‘Big Five’ [103], [104], comprising extraversion (propensity to seek and enjoy others’ company), agreeableness (trust and cooperation in social interactions), conscientiousness (attention to detail and focus), neuroticism (tendency to feel negative emotions) and openness (creativity, intellectual curiosity and willingness to seek new experiences). These traits are temporally stable within individuals [105] and are found in both sexes, all races and across cultures [106]. In addition, the State–Trait Anxiety Inventory [107], specifically the trait subscale, was used to determine the participants’ general tendency to feel anxious. Participants were also scored for two subscales (social skill and communication) from the Autism Spectrum Quotient, which measures the degree to which any neurotypical adultlies on the continuum between normality and autistic-like behaviours [108]. Social network size was evaluated based on the number of people in their two innermost layers, approximately corresponding to those individuals contacted on a weekly and monthly basis respectively [109].

Detailed dietary habits of the participants were assessed with the EPIC-Norfolk Food Frequency Questionnaire [110]. The associated software, Food Frequency Questionnaire EPIC Tool for Analysis [111], was used to estimate total calorie consumption and intake of food groups, macronutrients, minerals and vitamins (56 variables in total). Food frequency questionnaires with more than ten missing entries were excluded in accordance with the guidelines.

### 2.4 Multiple regression analyses of bacterial abundances

Microbiome count data are often overdispersed, with a high proportion of zeros [112], [113] and heavily right-skewed [114]. This is because there are relatively few taxa that are shared by the majority of samples, since most taxa are rare and only detected in a small proportion of samples [112]. Given this overdispersion, count data cannot be normalized via transformation and so negative binomial regression was conducted with genus abundance as the response variable (or zero-inflated negative binomial regression in cases where the count data contained an excess of zeros). When modelling microbiome data, it is necessary to account for variation in sequencing read depth between samples. However, rarefying count data or transforming it into proportions can lead to a high rate of false positives and is not recommended for analyses of taxon abundance [115]. Instead, the logarithm of the total read number was included as an offset in the regression models, thereby preserving statistical power by retaining all the count data [112], [116].

As opposed to the majority of microbiome studies which only conduct bivariate correlations between variables and microbial taxon abundances, key variables known to influence the gut microbiome were included in the regression models to avoid potential confounding effects. These variables were sex [117], [118], [119], [120], age [117], [121], body mass index [117], birth delivery mode [120], [122], [123], type of infant feeding method [118], [120], [124], whether participants had been treated with oral antibiotics within the last six months (since studies suggest the gut microbial community largely recovers after this time [125], [126]), whether they suffered from a gut condition [127], [128] and if they took probiotic supplements [36], [129] (though evidence is somewhat lacking as to the degree to which probiotic supplementation can alter faecal microbiota composition [130], [131]). These regression analyses could only be conducted on a subset of the population cohort (*n* = 261) who supplied the necessary data for all these variables.

The variables of primary interest were those relating to personality traits. Pairwise Kendall’s Tau-b correlation coefficients were computed to assess the correlation structure of all variables in the study, revealing considerable intercorrelation between extraversion, social skill and communication and also between neuroticism, anxiety and stress, as expected from the literature [132], [133]. To avoid multicollinearity in the regression models, two new variables were therefore created by summing the z-scores for the individual variables: sociability (a combined score of an individual’s extraversion, social skill and communication) and neurotic tendencies (a combined score of an individual’s neuroticism, anxiety and stress). These two new variables were used in the regression analyses since social behaviour, anxiety, stress and depressive-like behaviours are the main behavioural traits that have been linked to the gut microbiome [134].

Negative binomial regression analyses were conducted using the R package VGAM to model microbiome count data. All statistical analyses in this study were performed using R 3.2.3 software [135]. An extensive literature review was carried out to identify putative taxa that have been associated with behavioural or psychiatric traits in animal research or human populations. Genera that showed a significant association in at least two independent studies (Table 1) were then targeted in the regression analyses. Genus-level count data were used for the regression models since 16S rRNA sequencing provides an estimated 96% accuracy for genus identification [136] but has limited phylogenetic resolution at the species level [137].

### 2.5 Analyses of microbiome diversity and community composition

Analyses were also conducted to determine how the overall compositional profile of the microbiome, measured with alpha and beta diversity metrics, was related to the study variables. Alpha diversity is the ecological diversity of a single sample, taking into account the number of different taxa and their relative abundances, while beta diversity measures differences in microbial community composition between individuals [138]. The R package phyloseq was used to determine richness and alpha diversity at the genus level [139]. Since the choice of diversity index can significantly influence results [140], both Shannon’s diversity index and the inverse Simpson’s diversity index were calculated (the inverse of Simpson’s index being more intuitive since its value increases as diversity increases). While these two diversity indices take into account both richness and evenness, Shannon’s diversity index is more sensitive to changes in abundance of rare taxa, whereas the inverse Simpson’s diversity index gives more weight to common taxa [141].

Since the correlation between sequencing depth and diversity was not negligible, microbiome count data were rarefied prior to diversity analyses by randomly sampling counts without replacement such that all samples had an equal number of total counts. Although rarefying the count data was avoided for the regression models, it is still considered a useful normalization technique when conducting diversity analyses as uneven sequence counts across samples can significantly impact diversity estimates [113], [116]. In terms of alpha diversity, associations between each variable and either diversity or richness were measured using Kendall’s Tau-b correlation coefficient. This method is appropriate since it is a non-parametric measure of correlation and also accounts for tied values.

To assess beta diversity, permutational multivariate analysis of variance (PERMANOVA) was conducted to determine which variables significantly affect the overall composition of the gut microbiome. Distance matrices were computed using the distance function in the R package phyloseq [139] to assess the pairwise similarity of gut microbiome composition between individuals. The Bray–Curtis index provides a quantitative measure of dissimilarity between microbial communities, while the Jaccard index is qualitative since it only takes into account presence versus absence of taxa and not their abundances [116]. PERMANOVA was carried out on both Bray–Curtis and Jaccard distance matrices using the adonis function from the R package vegan with 1000 permutations. This function estimates the variance in the distance matrix that is attributable to the variable of interest, that is, the extent to which each variable may explain differences between individuals in microbial community composition. The *P* values from these analyses were then adjusted for multiple comparisons using the Benjamini–Hochberg method to control for the false discovery rate (FDR), with *P*_adj_ < 0.1 considered statistically significant.

## 3 Results

### 3.1 Summary statistics

**Fig. 2:**
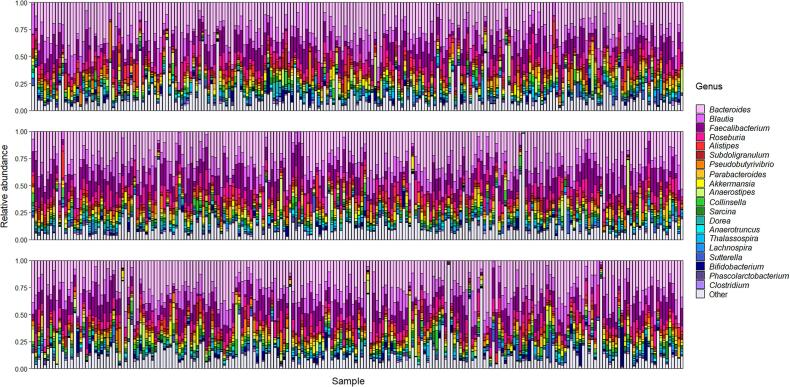
Stacked bar plots summarizing gut microbiome composition at genus level for the total participant cohort. Microbiome data are plotted showing relative abundance of the top twenty most abundant genera across all samples, with the remaining genera grouped into ‘Other’. The twenty genera in the key are ordered from most to least abundant.

Gut microbiome composition differed markedly between individuals (Fig. 2), as has repeatedly been shown in human populations [142]. Out of all the genera detected across the gut microbiome samples, the top twenty most abundant genera showed close correspondence with previous research [143]. *Bacteroides* was the most abundant genus, as found in other studies [144], [145]. There was also considerable variation among the study population in the variables assessed in the questionnaire (Tables S1 and S2). The majority of study variables from the questionnaire showed little to modest intercorrelation (Fig. S1), though as expected there was a high degree of correlation between the nutrient variables assessed in the food frequency questionnaire (Fig. S2).

### 3.2 Regression models of bacterial abundances

**Fig. 3:**
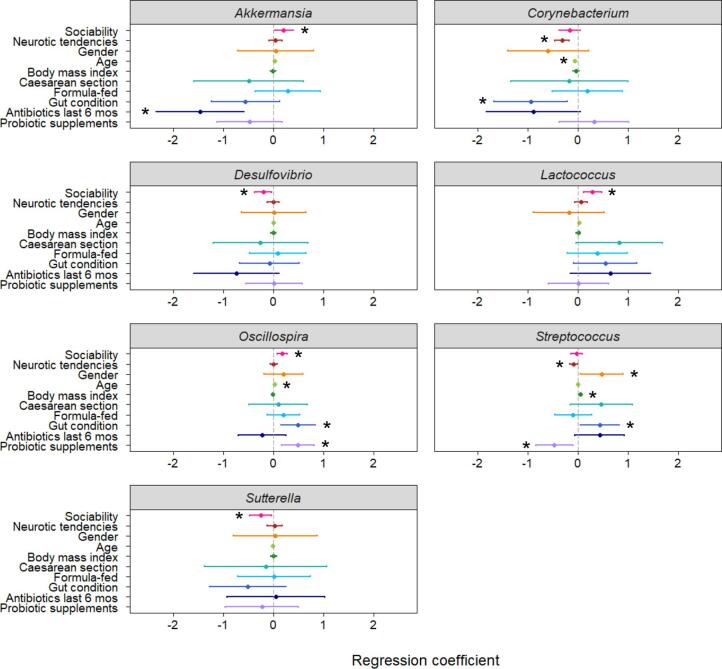
Coefficient plots from regression models predicting abundances of genera in the human gut microbiome. Asterisks denote significant predictors of genus abundance where *P* < 0.05 and bars indicate 95% confidence intervals. The key variables of interest for this study were those relating to behavioural traits, namely sociability (extraversion, social skill and communication) and neurotic tendencies (neuroticism, anxiety and stress), while other variables were also included to avoid potential confounding effects. A positive relationship with gender indicates a higher abundance of the genus in females. Plots displayed here depict genera whose abundances were significantly related to differences in behavioural traits (for remaining regression coefficient plots see Fig. S3).

Negative binomial regression analyses revealed that the abundances for seven of the 23 genera were significantly predicted by individual variation in behavioural traits (Fig. 3; Table S3). Sociability (a combined measure of the participant’s extraversion, social skill and communication) was a positive predictor of genus abundance for *Akkermansia* (*P* = 0.038), *Lactococcus* (*P* = 0.002) and *Oscillospira* (*P* < 0.001), and a negative predictor of the abundances of *Desulfovibrio* (*P* = 0.019) and *Sutterella* (*P* = 0.028). The variable assessing neurotic tendencies (a combined measure of neuroticism, anxiety and stress) negatively predicted genus abundance for *Corynebacterium* (*P* < 0.001) and *Streptococcus* (*P* = 0.029). Table 2 compares these bacterial genera found to be significantly related to behavioural traits with the results of previous studies on these genera.

**Table 2:** Bacterial genera found to be significantly related to behavioural traits in this study compared with previous research findings extracted from Table 1. For genera significantly related to sociability in this study, it is most relevant to compare these findings to those from studies on sociability or autism (i.e. if genus abundance is higher in sociable individuals, it may be expected to be lower in autism). For genera related to neurotic tendencies it is most relevant to compare these findings to those from studies on stress and depression. Results from previous studies that are clearly in the opposite direction to the findings in this study are marked in brackets.

Genus	Change in abundance	Behavioural trait/psychiatric condition	Study subject	References
*Akkermansia*	↓	Autism	Children	[52]
	[↑]	Autism	Children	[53]
	↓	Stress	Mice	[54], [55]
	↑	Sociability	Adults	This study
*Corynebacterium*	[↑]	Autism	Children	[56]
	↓	Stress	Rats	[83]
	↓	Neurotic tendencies	Adults	This study
*Desulfovibrio*	↑	Autism	Children	[60], [80]
	↓	Sociability	Adults	This study
*Lactococcus*	↓	Autism	Children	[53], [60]
	↑	Sociability	Adults	This study
*Oscillospira*	↓	Autism	Children	[53]
	↑	Sociability	Mice	[94]
	↓	Stress	Mice	[55], [94], [95]
	↑	Sociability	Adults	This study
*Streptococcus*	↓	Autism	Children	[53], [60]
	[↑]	Depression	Adults	[82]
	↓	Neurotic tendencies	Adults	This study
*Sutterella*	↑	Autism	Children	[52], [65], [96], [97]
	[↓]	Autism	Children	[75]
	↑	Stress	Mice	[55]
	↓	Sociability	Adults	This study

There were also notable results in terms of the possible confounding factors included in the models. Age and body mass index were common predictors of abundance, significantly predicting the abundances of eight and seven genera, respectively. In contrast, birth delivery mode and infant feeding method had little influence on the abundances of most genera. Delivery mode was only a significant predictor of the abundance of one genus, *Flavonifractor*, with a higher abundance in individuals born by caesarean section. Formula feeding during infancy was a positive predictor of *Lactobacillus* abundance, in agreement with previous research findings [146]. Surprisingly, abundances of only three of the 23 genera were significantly altered by antibiotic treatment in the past six months, indicative of the differential effects antibiotics can have on bacterial taxa. Three genera, *Lactobacillus*, *Prevotella* and *Streptococcus*, were more abundant in females, which is consistent with previous findings for *Lactobacillus* [120], [147].

### 3.3 Microbiome diversity

**Fig. 4:**
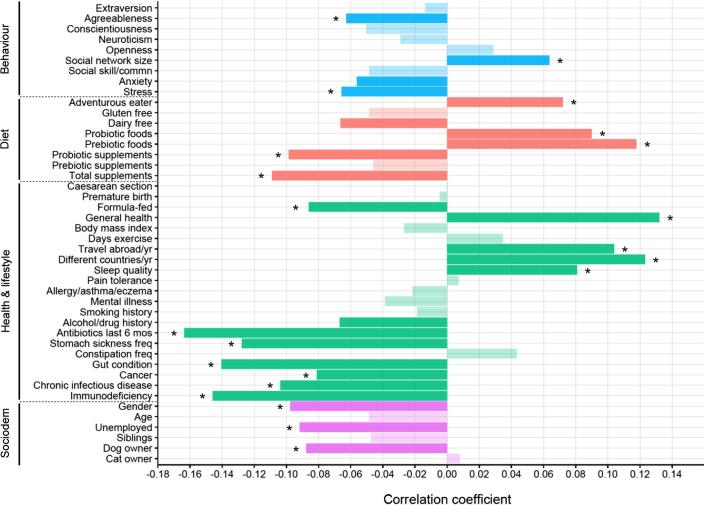
Bar plot showing results of Kendall’s Tau-b correlation analysis between gut microbiome diversity and the study variables. Opaque shading indicates a significant correlation at FDR < 0.1 and asterisks denote significance also at FDR < 0.05. The negative relationship with gender reflects a lower diversity in females compared with males. Plot depicts results with Shannon’s diversity index (for results using the inverse Simpson’s diversity index and genus richness see Table S4).

Overall, 25 of the 44 study variables were significantly related to gut microbiome diversity (*P*_adj_ < 0.1), as measured by Shannon’s diversity index, with the majority of these (22 variables) also retaining significance at FDR < 0.05 (Fig. 4; Table S4). For the primary variables of interest relating to behaviour, both stress (*P*_adj_ = 0.045) and anxiety (*P*_adj_ = 0.077) were negatively correlated with Shannon’s diversity index, and agreeableness was also negatively related (*P*_adj_ = 0.048). There was a positive correlation between Shannon’s diversity index and social network size (*P*_adj_ = 0.043), such that individuals with a larger social network tended to have a more diverse gut microbiome.

In terms of the other variables in the study, more adventurous eaters and those who ate more foods with naturally occurring probiotics (fermented foods) or prebiotics (non-digestible fibre) tended to have a more diverse gut microbial community. In contrast, consumption of probiotic supplements was significantly correlated with lower diversity. Variables relating to ill health were generally associated with reduced diversity and, not surprisingly, the strongest negative relationship was with antibiotic treatment. Adults who were formula-fed as infants had a significantly less diverse microbiome compared with those that were breast-fed. There was also evidence that females had a lower gut microbiome diversity than males. Diversity was not related to exercise frequency or body mass index, while the strongest positive correlation was with general health. Microbiome diversity was positively correlated with travel, while it was negatively related to dog ownership and also unemployment.

Comparing the correlation results of Shannon’s diversity index and the inverse Simpson’s diversity index revealed good consistencybetween the two indices (Table S4). Most of these relationships were also present when correlating the variables with the observed number of genera (Table S4), though as expected there were some differences between the results since this is a measure of community richness rather than diversity. Notably, there was a significant negative correlation between conscientiousness and genus richness (*P*_adj_ = 0.014), which was in the same direction as the non-significant trend with diversity.

With respect to nutrient intake estimated from the food frequency questionnaire, the consumption of fruit and cereals, and therefore fibre (and more generally carbohydrates), was positively related to diversity (Table S5). This was in accordance with previous findings that the metabolism of carbohydrates, particularly fibre, promotes microbial diversity [148], [149], [150]. Diversity was also significantly correlated with the dietary abundance of a range of minerals and vitamins (Table S5). There was little relationship between genus richness and nutrient intake with the exception of fish consumption which was positively related.

### 3.4 Microbial community composition

**Fig. 5:**
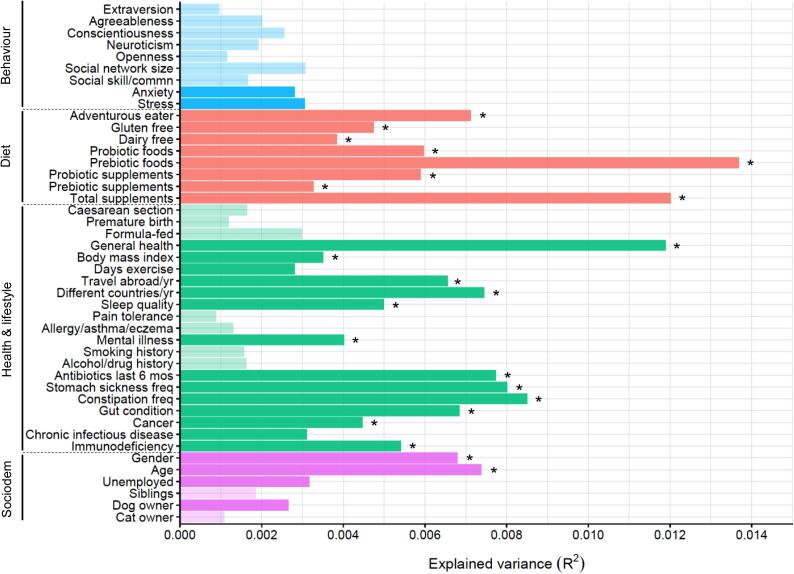
Bar plot showing explained variance in gut microbiome composition in relation to the study variables. Opaque shading indicates variables explaining significant inter-individual variation at FDR < 0.1 and asterisks denote significance also at FDR < 0.05. Plot depicts results using the Bray–Curtis distance matrix (for results with the Jaccard distance matrix see Table S6).

Differences in the composition of the gut microbiome, as quantified by the Bray–Curtis index, were significantly related to 28 of the 44 variables (*P*_adj_ < 0.1), with most of these (22 variables) retaining significance at FDR < 0.05 (Fig. 5; Table S6). While the *P* values reported below refer to results with the Bray–Curtis index, the same variables were also significant when using the Jaccard index to measure differences in microbial community composition (Table S6).

Regarding behavioural traits, anxiety (*P*_adj_ = 0.094) and stress (*P*_adj_ = 0.052) were significantly related to an altered microbiome composition. Another factor influencing composition was whether the participant suffered from a mental illness (*P*_adj_ = 0.020). The majority of variables assessing health and lifestyle were also significantly related to composition, whereas there was no effect of early-life events (mode of delivery, premature birth and infant feeding). While exercise frequency did not significantly influence diversity, it did affect overall composition of the gut microbiome, as has been shown in other studies [151]. As was the case with diversity, unemployment was a significant factor influencing microbial community composition. Composition also differed by age and gender, in line with previous research [121].

All the main dietary variables were significantly related to composition (Fig. 5). Both gluten-free and dairy-free diets were associated with an altered microbial community, in accordance with other research findings [152], [153], [154]. The consumption of naturally occurring prebiotic fibre showed the strongest relationship with gut microbiome composition. In terms of the results of the food frequency questionnaire, consumption of fruit, vegetables and cereals, and therefore the total intake of carbohydrates including fibre, were all significantly related to microbiome composition, as well as certain minerals and vitamins (Table S7).

## 4 Discussion

Investigations of the microbiome–gut–brain axis have predominantly been conducted in animal models or clinical populations, with few studies exploring whether these findings are translatable to the general population. Here both gut microbiome composition and diversity were found to be related to differences in personality. This is the first time that abundances of microbial genera have been modelled with respect to human personality, while also rigorously controlling for the possible confounding effects of key variables known to influence the gut microbiome. The regression results revealed that abundances of particular genera are significantly related to behavioural traits, suggesting that studying the gut microbiome may be relevant to understanding variation in human personality. People with larger social networks were also found to have more diverse microbial communities,indicating that social behaviour may promote diversity of the human gut microbiome. By contrast, lower diversity was associated with increased levels of stress and anxiety, and these traits were also related to differences in overall composition of the microbial community. In addition, the intercorrelation analysis (Fig. S1) revealed that people who ate more foods with naturally occurring probiotics or prebiotics had significantly lower levels of anxiety, stress and neuroticism and were also less likely to suffer from a mental illness, but this relationship was not found in those consuming probiotics or prebiotics in supplement form. Consistent with this, a previous study reported that females consuming more fermented foods (a source of natural probiotics) had fewer symptoms of social anxiety [155] and similarly a traditional Japanese diet rich in fermented soy products has been linked to reduced depressive symptoms [156].

The significant relationships between personality traits and the abundances of particular genera targeted in the regression analyses were largely in the expected direction based on studies in humans with psychiatric conditions and in laboratory animals (Table 2). The genera *Akkermansia*, *Lactococcus* and *Oscillospira* were found to be more abundant in individuals with a higher sociability score. In accordance with these results, previous studies in children have reported a reduction in *Lactococcus* and *Oscillospira* in autism, while one study found decreased abundance of *Akkermansia* (Table 2). *Oscillospira* has also been positively related to sociability in mice (Table 2). Interestingly, both *Akkermansia* and *Oscillospira* are associated with good health; *Akkermansia* has anti-inflammatory properties and there is some evidence it may be protective against metabolic disorders [157] while lower levels of *Oscillospira* are linked to inflammatory disease [158]. Two genera, *Desulfovibrio* and *Sutterella*, were more abundant in less sociable individuals. This is in line with those studies finding that their abundances are also elevated in people suffering from autism (Table 2) and in fact, it has been hypothesized that *Desulfovibrio* species may play an important role in the pathophysiology of autism [159]. The variable measuring neurotic tendencies was a significant negative predictor of the abundances of *Streptococcus* and *Corynebacterium*. Therefore, individuals that were more neurotic tended to have lower levels of these genera. Since higher neuroticism is a known risk factor for developing depression [160], this result is in agreement with a study in a rat model of stress-induced depression reporting a reduction in *Corynebacterium* (Table 2). However, in contrast to the findings here, *Streptococcus* has previously been found to be more prevalent in adults suffering from depression (Table 2). Although *Streptococcus* species are part of a healthy microbiome, members of this genus can be opportunistic pathogens [161].

The results of this study suggest that some bacterial genera may be more strongly linked to behaviour and, in light of my findings, perhaps future research may benefit from investigating the effects of the genera *Akkermansia*, *Desulfovibrio*, *Lactococcus*, *Oscillospira* and *Sutterella* on social behaviour and autistic-like symptoms in animal models, with a view to developing potential new therapies for autism. Notably, not all of the 23 genera targeted in these regression analyses were significantly predicted by behavioural traits (Fig. S3; Table S3), though this was expected given the somewhat inconsistent results reported in the literature (Table 1). In particular, despite extensive findings from animal studies that species belonging to *Bifidobacterium* and *Lactobacillus* can alleviate symptoms of anxiety, stress and depression, and also improve social behaviour [42], [162], [163], [164], [165], [166], [167], [168], [169], abundances of these genera were not significantly related to either sociability or neurotic tendencies in this study. This may be because these regression analyses were conducted at the genus level, yet there is some evidence that the behavioural effects of *Bifidobacterium* and *Lactobacillus* may be species-specific and even strain-specific [42], [163].

Interpreting results from this study also requires consideration of the bidirectional interactions between the gut microbiome and behaviour. As well as the behavioural effects of gut microorganisms, behaviour can in turn shape the composition of the gut microbiome. For example, the gut microbiome can affect the stress response and stress also disrupts the gut microbiome [6], [15]. In addition, there is growing evidence that how an animal interacts socially can significantly influence its gut microbial community, whose composition is the product of microbial immigration and competition throughout life via transmission of microorganisms, primarily through the faecal–oral route [170]. Research in wild primate populations has revealed that social contact shapes gut microbiome composition [171], [172], [173], [174], [175], [176] and studies in humans find that we share gut microbiota with household members [121], [177], [178], [179], [180], [181]. A key research avenue is untangling the contributions of specific microorganisms to social behaviour versus the influence of social behaviour on microbial colonization. In at least some cases, microbial abundances may differ in relation to autistic traits or sociability because certain taxa are better adapted for transmission between hosts. Notably, the genus *Oscillospira*, whose abundance was positively predicted by sociability in this study, has also been found to be positively related to host density in a wild animal population [182]. It is important to bear in mind, therefore, that some genera may be efficiently transmitted socially, rather than having a causal effect on social behaviour. Indeed, *Oscillospira* belongs to the family Ruminococcaceae, many of which are intestinal spore-forming bacteria, thereby facilitating transmission between hosts [183]. In fact, numerous members of the microbiota are well adapted for transmission and it is estimated that at least half the bacterial genera in the gut are able to produce resilient spores adapted for survival and dispersal [183].

The evidence of social transmission of microorganisms is particularly relevant to the finding here that people with larger social networks tend to have a more diverse gut microbiome. Indeed, animal studies have revealed that social interactions are positively associated with microbiome diversity [172], [175], [182], [184]. For example, reduced social contact in a bumblebee colony results in lower gut microbiome diversity [184] and chimpanzees that interact more socially have a more diverse gut microbiome [172]. In fact, horizontal transmission of gut microorganisms through chimpanzee social interactions appears to play a greater role than vertical transmission from the mother in shaping gut microbiome composition [172]. This suggests that although the mother provides the primary inoculum for the newborn’s microbiome, the composition of the microbiome through the individual’s lifetime may be more strongly influenced by social interactions. The relationship between gut microbiome diversity and human social networks has not previously been explored but the positive relationship found here suggests that social interactions may also influence the microbiota of human societies. Interestingly, a study of gut microbiome composition and temperament in infants reported an association between gut microbiome diversity and sociability [84]. By maintaining diversity of the gut microbial community, social transmission of microorganisms may benefit host health in numerous ways [170]. For example,diversity may help to promote stability and resilience of the gut microbiome [142], and is often linked to good health, though this is not always the case [140]. Diversity can provide resistance against infection [185], [186], [187], [188], improve immune function [170] and may reduce the risk of allergies [189]. It is well known that people with more social ties are healthier and live longer, and reduced inflammation is postulated to play a role in this relationship [190], [191]. It is interesting therefore to speculate whether the microbiome may mediate this association between social integration and health, particularly since it is a key regulator of the host immune response [192].

Participants who were more stressed or anxious, and also those reporting poorer sleep quality, tended to have both a less diverse gut microbiome and an altered composition. In line with these results, a recent study found that anxiety status was related to stool consistency, suggesting that anxiety may be associated with differences in gut microbiome composition, perhaps through inducing dysbiosis [193]. Depression has also been associated with differences in microbial community composition [194]. In addition, it has previously been shown in animal studies that stress not only alters the abundances of various microbial taxa but also reduces the diversity of the gut microbial community [61], [195]. In fact, part of the reason why individuals with a larger social network have a more diverse microbiome may be because social support can help buffer the adverse effects of stress on diversity [196]. However, social network size was not significantly correlated with stress in this study (Fig. S1) and the positive relationship between network size and microbiome diversity is consistent with evidence from animal populations that members of the microbiota are transmitted through interactions with the social environment. Further research should attempt to validate this finding and investigate the various microbial transmission routes and their relative importance. Indeed, modern lifestyle choices are geared towards preventing pathogen transmission but maybe we should instead consider ways to promote the spread of beneficial microorganisms [170]. Notably, the negative relationship found here between conscientiousness and genus richness of the gut microbiome may be because conscientious people are more likely to engage in hygienic behaviour [197], which may result in a smaller number of microbial genera inhabiting the gut. The observed relationship between sleep quality and gut microbiome composition and diversity may be due to its intercorrelation with stress, anxiety and neuroticism (Fig. S1) but may also partly reflect the known relationship between the gut microbiome and host circadian rhythms [198].

Although the focus of this research was on personality traits, there are also other novel findings of considerable interest from this study. People who travelled frequently or visited more countries tended to have a more diverse gut microbiome, suggesting that our interaction with the environment does play a considerable role in influencing our gut microbial community. This increased diversity may also partly reflect the different foods people tend to eat when travelling. However, holidays abroad are also positively associated with the prevalence of antibiotic resistance genes in the gut [199]. This suggests that although travelling may increase the taxonomic diversity of the gut, it may also increase the risk of acquiring antibiotic resistance. Another interesting result not previously shown was that more adventurous eaters also had a greater gut microbiome diversity, supporting the idea that microbiome health may be improved through a diverse diet [200]. This finding is particularly pertinent given the increasingly restrictive dietary habits of Western cultures and is also in agreement with a recent study on a wild primate population which reported a positive association between dietary diversity and microbiome richness [175].

Diversity of the gut microbiome was also related to the amount of food people consumed containing natural probiotics and prebiotics. Indeed, fermentation has been practised by human civilization for over 9000 years [201] and is traditionally used in many cultures in the production of foods such as cheese, yogurt, sauerkraut and kimchi. From an evolutionary perspective, fermented foods may represent anadaptation passed on through generations, given their potential beneficial effects on health. However, probiotic supplementation was significantly associated with reduced microbiome diversity. A likely explanation for this finding is that people with reduced microbiome diversity (for example, due to a course of antibiotics or gut dysbiosis) may be more inclined to take probiotic supplements. Indeed, while there was no evidence of a relationship between probiotic consumption and either antibiotic treatment or general health, probiotic supplementation was positively correlated with having a gut condition (Fig. S1).

There was no significant effect of delivery mode, premature birth or infant feeding practice on microbial community composition, though these factors may still influence establishment of the microbiome during infancy, with potential consequences for development of the immune system. However, regression results revealed that one genus, *Flavonifractor*, was differentially abundant in those born vaginally compared with caesarean section, with a higher abundance in individuals born by caesarean section. Notably, *Flavonifractor* has also been found to be more abundant in infants with food allergies [202] and birth by caesarean section is often linked to an increased risk of allergies [203]. Although there is some evidence that birth by caesarean section can affect the diversity and community composition of the infant gut microbiome [120], [122], [123], other studies find that delivery mode does not significantly influence development of the microbiome [204], [205], [206] and no lasting differences (except *Flavonifractor* abundance) were detected here in the adult population. However, individuals that were formula-fed as infants did have a lower gut microbiome diversity. This is the first time the effect of infant diet has been explored with respect to the adult gut microbiome. Most research in infants actually finds that formula feeding is associated with increased diversity compared to breastfeeding [124], but results of a two-year longitudinal study have shown that while formula-fed infants in their first year of life may have higher gut microbiome diversity, breast-fed infants tend to have higher diversity between one and two years of age [123].

Other notable results include females having a significantly lower gut microbiome diversity compared to males. This contrasts a previous human study which found no significant differences in diversity between the sexes [207] but is in the same direction as recent research in a wild baboon population [174]. As expected, antibiotic treatment was associated with reduced diversity of the microbiome and an altered composition, in accordance with other findings [126], [208], [209], [210], [211]. Interestingly, fish consumption was positively associated with genus richness of the gut microbiome, perhaps due to exposure to different bacterial genera inhabiting marine ecosystems. Somewhat surprisingly, dog ownership was linked to lower diversity, which contrasts the finding that infants living with household pets tend to have a more diverse gut microbiome [212]. The reduced microbiome diversity of people in unemployment may partly be a reflection of socioeconomic status which can influence health through lifestyle behaviours, diet, access to medical care and psychosocial factors such as stress [213]. With respect to other comprehensive population studies of the human gut microbiome [204], [194], the results here replicate previous findings that age, gender, body mass index, sleep, constipation, gut conditions, antibiotic treatment and consumption of fruit, vegetables and cereals are related to gut microbiome variation.

As this study was cross-sectional, causation cannot be proved, particularly given the bidirectional nature of the microbiome–gut–brain axis. As discussed, gut bacteria can affect behaviour and behaviour can in turn influence the composition of the gut microbiome. Despite this limitation, the findings here represent an important step in understanding the relationships between the gut microbiome and personality traits, and the potential consequences for mental health. However, since this is one of the first studies linking the gut microbiome to personality, further research would help to confirm the reproducibility of these findings. In addition, it should be borne in mind that there are numerous factors that can affect personality. In particular, genetics account for approximately 50% of variation in personality, while a range of environmental factors contribute to the remaining variation [1]. Since both host genetics and the environment are known to affect microbiome composition [214], it may be that some of the effects of genes or the environment on personality are via their influence on the microbiome.

A key benefit of this study is that rather than examining the expression of extreme traits, as seen in psychiatric disorders, this research focused on behavioural variation in the general population. The findings reported here challenge the results of a recent study concluding a lack of significant relationships between psychiatric measures in healthy humans and microbiome composition and diversity [215]. However, that research was based on a comparatively small sample size and only involved female participants. Given the very exploratory nature of their study, the researchers FDR-corrected their *P* values for almost 4000 hypotheses which may have led to numerous false negative results. As opposed to their conclusions that the gut microbiome may only be relevant in cases of psychiatric illness, my findings suggest that the microbiome may also be related to personality traits in the healthy population. Indeed, the majority of the results were replicated when anyone suffering from a psychiatric condition was excluded from the dataset, showing that my findings were not driven by those scoring towards the extreme end for the personality traits (Tables S8 to S10). The only notable exception was for the beta diversity analyses, where stress and anxiety were no longer significantly associated with differences in the overall community composition of the gut microbiome. The majority of the regression results were also replicated, although the positive relationship between sociability and *Akkermansia* abundance was not detected. Additionally, there was evidence in this dataset that *Blautia* was less abundant in more sociable individuals (*P* = 0.043) and *Prevotella* was less abundant in more neurotic individuals (*P* = 0.002).

In conclusion, differences in gut microbiome composition and diversity are shown to be linked to personality traits in the general population. The results of this study add a new dimension to our understanding of personality and are in line with accumulating evidence that the gut microbiome can influence the central nervous system in humans, with effects on behaviour. Such findings may inform the development of probiotic or prebiotic therapies to help improve mood and treat conditions such as autism, anxiety and depression. Discovering new and effective interventions for mental health conditions is of pressing concern, given the declines in psychological health of our modern society. Finally, it is pertinent to reflect on the ways in which our modern-day living may provide a perfect storm for dysbiosis of the gut. We lead stressful lives with fewer social interactions and less time spent with nature, our diets are typically deficient in fibre, we inhabit oversanitized environments and are dependent on antibiotic treatments. All these factors can influence the gut microbiome and so may be affecting our behaviour and psychological well-being in currently unknown ways.
