# Supplementary material for: Gut microbiome composition and diversity are related to human personality traits
Source: Hum Microb J. 2020 Mar;15:None. doi: 10.1016/j.humic.2019.100069 (PMC8336012; doi:10.1016/j.humic.2019.100069)
Supplement: Supplementary data 1 [file mmc1.pdf]

Supplementary Information

Supplementary Figures

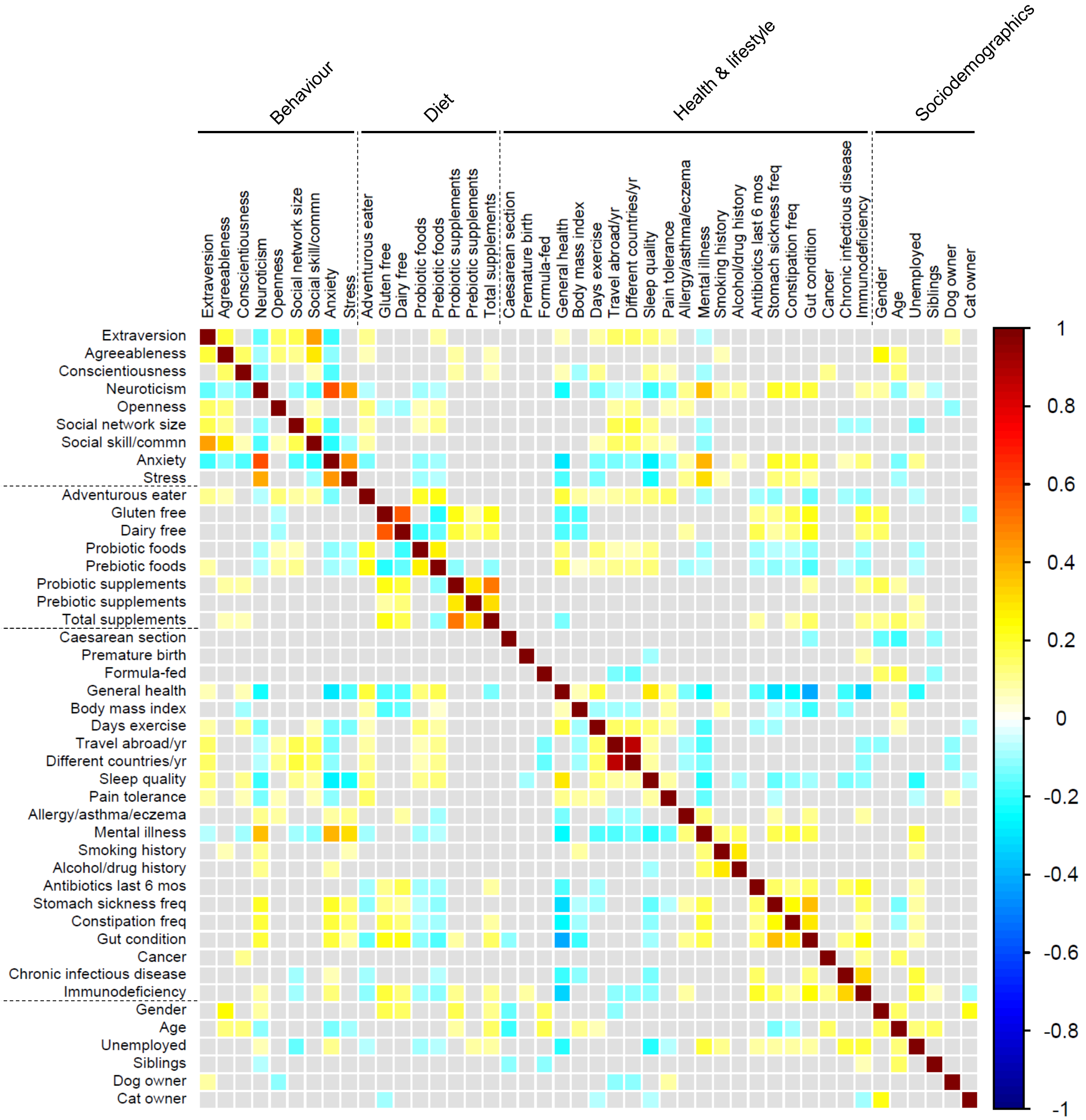

Fig. S1. Intercorrelation plot for the study variables, divided into the four categories: behaviour, diet, health and lifestyle, and sociodemographics. Plot depicts strength and direction of pairwise correlations, as measured by Kendall's Tau-b correlation coefficient. Grey squares denote associations that were not statistically significant at FDR < 0.1.

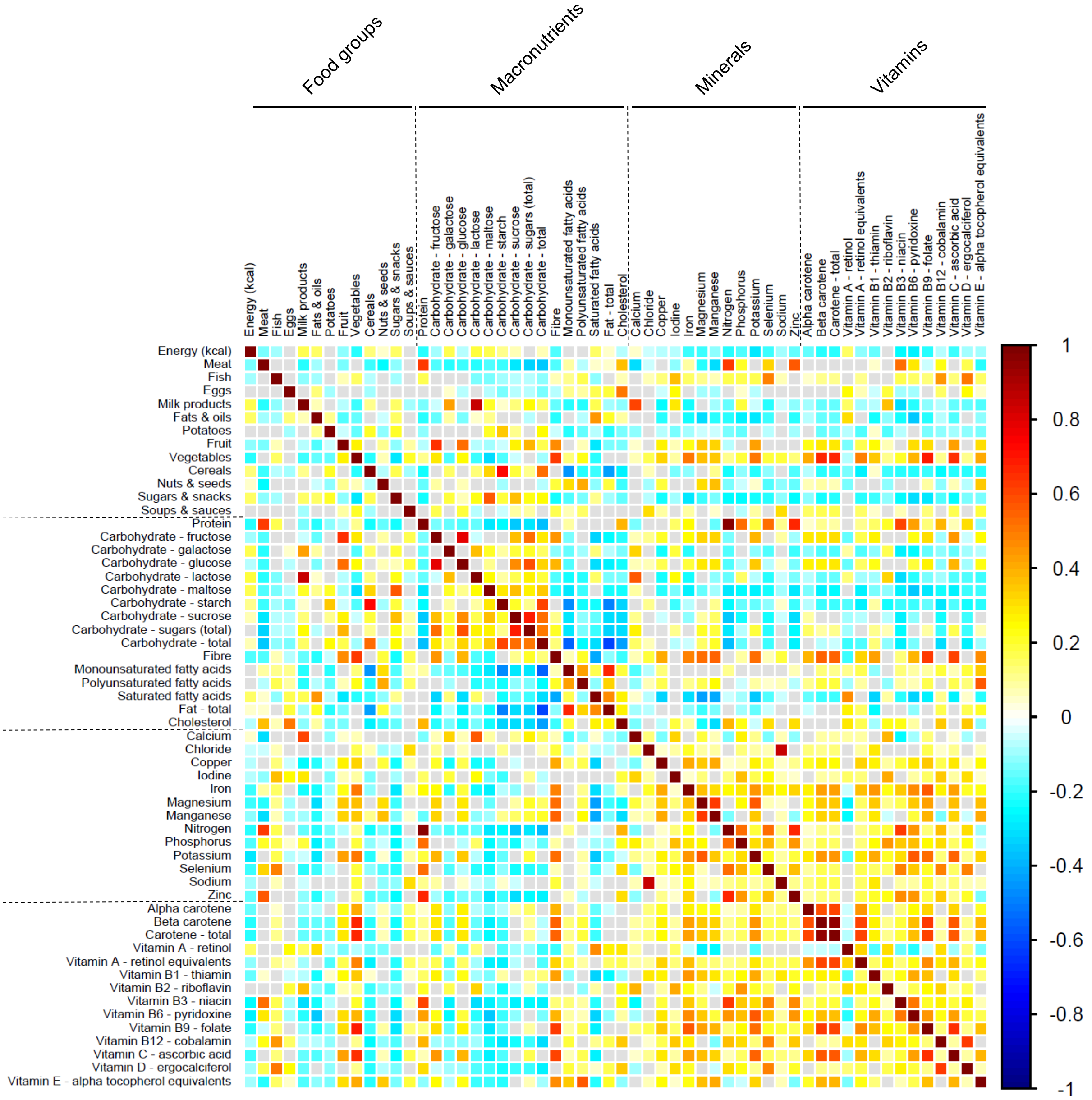

Fig. S2. Intercorrelation plot for the nutrient variables, divided into the four categories: food groups, macronutrients, minerals and vitamins. Plot depicts strength and direction of pairwise correlations, as measured by Kendall's Tau-b correlation coefficient. Grey squares denote associations that were not statistically significant at FDR < 0.1. Note that the quantity of each nutrient variable was calculated relative to estimated total calorie intake prior to the intercorrelation analysis.

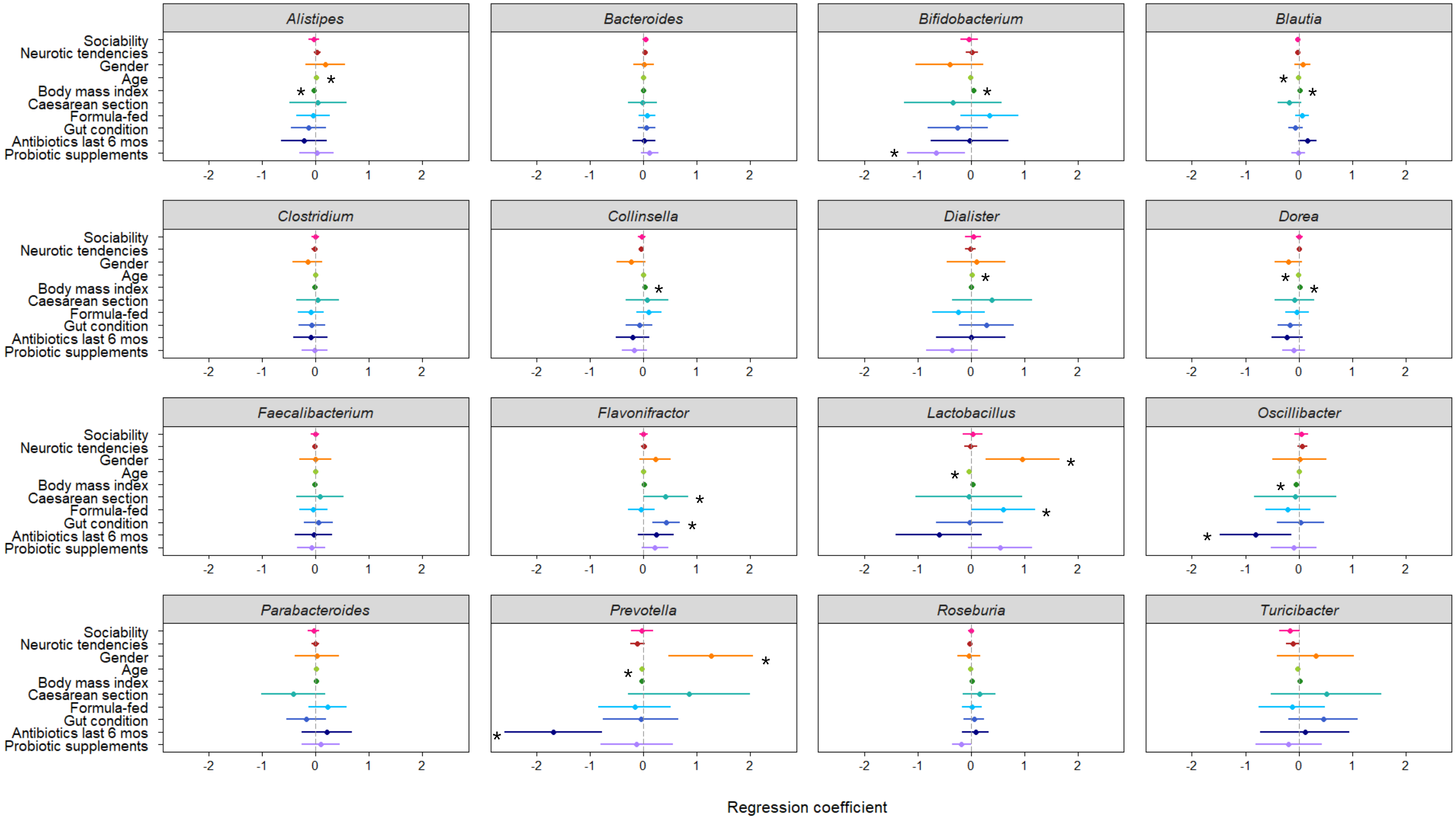

Fig. S3. Coefficient plots from regression models predicting abundances of genera in the human gut microbiome. Asterisks denote significant predictors of genus abundance where $P < 0.05$ and bars indicate 95% confidence intervals. The key variables of interest for this study were those relating to behavioural traits (sociability and neurotic tendencies), while other variables were also included to avoid potential confounding effects. A positive relationship with gender indicates a higher abundance of the genus in females. Plots displayed here depict genera whose abundances were not significantly related to differences in behavioural traits (for significant results see Fig. 3).

Supplementary Tables

Table S1. Summary statistics for continuous variables. Mean, standard error and range for each study variable are given. For the questionnaires assessing personality phenotype, the maximum possible range of scores are 10 to 50 for the 50-item International Personality Item Pool Inventory, 20 to 80 for the State–Trait Anxiety Inventory and 0 to 20 for the Autism Spectrum Quotient (social skill and communication).

Table S2. Data summary for binary variables. Values represent percentage of participants who answered 'yes' to each variable.

Table S3. Output from regression models predicting abundances of genera in the human gut microbiome. *P* values highlighted in green denote significant predictors of genus abundance where $P < 0.05$. Note that two *P* values are given for zero-inflated negative binomial regression models, with the first *P* value indicating whether the variable predicts genus abundance, and the second *P* value indicating whether the variable predicts the presence versus absence of the genus. CL represents 95% confidence limit for each regression coefficient. A positive regression coefficient for gender indicates a higher abundance of the genus in females.

Table S4. Results of Kendall's Tau-b correlation analysis between gut microbiome diversity and study variables. Highlighted cells denote significant associations where $P < 0.05$ and $P_{adj} < 0.1$. Values in bold indicate $P_{adj} < 0.05$.

Table S5. Results of Kendall's Tau-b correlation analysis between gut microbiome diversity and nutrient variables. Highlighted cells denote significant associations where $P < 0.05$ and $P_{adj} < 0.1$. Values in bold indicate $P_{adj} < 0.05$.

Table S6. Results of PERMANOVA to test for an association between gut microbiome composition and study variables. PERMANOVA with 1,000 permutations was conducted on both Bray–Curtis and Jaccard distance matrices. Highlighted cells denote significant associations where $P < 0.05$ and $P_{adj} < 0.1$. Values in bold indicate $P_{adj} < 0.05$.

Table S7. Results of PERMANOVA to test for an association between gut microbiome composition and nutrient variables. PERMANOVA with 1,000 permutations was conducted on both Bray–Curtis and

Jaccard distance matrices. Highlighted cells denote significant associations where $P < 0.05$ and $P_{adj} < 0.1$. Values in bold indicate $P_{adj} < 0.05$.

Table S8. Output from regression models predicting abundances of genera in the gut microbiome, with sufferers of a psychiatric condition excluded from dataset. *P* values highlighted in green denote significant predictors of genus abundance where $P < 0.05$. Note that two *P* values are given for zero-inflated negative binomial regression models, with the first *P* value indicating whether the variable predicts genus abundance, and the second *P* value indicating whether the variable predicts the presence versus absence of the genus. CL represents 95% confidence limit for each regression coefficient.

Table S9. Results of Kendall's Tau-b correlation analysis between gut microbiome diversity and study variables, with sufferers of a psychiatric condition excluded from dataset. Highlighted cells denote significant associations where $P < 0.05$ and $P_{adj} < 0.1$. Values in bold indicate $P_{adj} < 0.05$.

Table S10. Results of PERMANOVA to test for an association between gut microbiome composition and study variables, with sufferers of a psychiatric condition excluded from dataset. PERMANOVA with 1,000 permutations was conducted on both Bray–Curtis and Jaccard distance matrices. Highlighted cells denote significant associations where $P < 0.05$ and $P_{adj} < 0.1$. Values in bold indicate $P_{adj} < 0.05$.
